# The METRIC-framework for assessing data quality for trustworthy AI in medicine: a systematic review

**DOI:** 10.1038/s41746-024-01196-4

**Published:** 2024-08-03

**Authors:** Daniel Schwabe, Katinka Becker, Martin Seyferth, Andreas Klaß, Tobias Schaeffter

**Affiliations:** 1 Division Medical Physics and Metrological Information Technology, Physikalisch-Technische Bundesanstalt, Berlin, Germany; 2 Department of Medical Engineering, Technical University Berlin, Berlin, Germany; 3 Einstein Centre for Digital Future, Berlin, Germany

**Keywords:** Scientific data, Health care, Statistics

## Abstract

The adoption of machine learning (ML) and, more specifically, deep learning (DL) applications into all major areas of our lives is underway. The development of trustworthy AI is especially important in medicine due to the large implications for patients’ lives. While trustworthiness concerns various aspects including ethical, transparency and safety requirements, we focus on the importance of data quality (training/test) in DL. Since data quality dictates the behaviour of ML products, evaluating data quality will play a key part in the regulatory approval of medical ML products. We perform a systematic review following PRISMA guidelines using the databases Web of Science, PubMed and ACM Digital Library. We identify 5408 studies, out of which 120 records fulfil our eligibility criteria. From this literature, we synthesise the existing knowledge on data quality frameworks and combine it with the perspective of ML applications in medicine. As a result, we propose the METRIC-framework, a specialised data quality framework for medical training data comprising 15 awareness dimensions, along which developers of medical ML applications should investigate the content of a dataset. This knowledge helps to reduce biases as a major source of unfairness, increase robustness, facilitate interpretability and thus lays the foundation for trustworthy AI in medicine. The METRIC-framework may serve as a base for systematically assessing training datasets, establishing reference datasets, and designing test datasets which has the potential to accelerate the approval of medical ML products.

## Introduction

During the last decade, the field of artificial intelligence (AI) and in particular machine learning (ML) has experienced unprecedented advances, largely due to breakthroughs in deep learning (DL)^1–5^ and increased computational power. Recently, the introduction of easy-to-use yet still extremely capable models such as GPT-4^6^ and Stable Diffusion^7^ has further expanded the technology to an even broader audience. The large-scale handling and implementation of AI^8^ into fields such as manufacturing, agriculture and food, automated driving, smart cities and healthcare has since shifted the topic into the centre of attention of not just scholars and companies but the general public.

The introduction of novel and disruptive technologies is typically accompanied by an oscillating struggle between exploiting technological chances and mitigation of risks. ML is proving to have great potential to improve many aspects of our lives^9–11^. However, the race for implementation and utilisation is currently outpacing comprehension of the technology. The complex and black box character of AI applications has therefore largely steered the public conversation towards safety, security and privacy concerns^12,13^. A lack of confidence of the general population in the transparency of AI prevents its utilisation for society and economic growth. It can lead to a slowed adoption of innovations in crucial areas and discourage innovators from unlocking the technology’s full potential. Hence, the demand for regulation (e.g., EU AI Act^14^, US FDA considerations^15^) as well as the need for an improved understanding of AI is ever increasing. This is of particular importance in the field of healthcare due to its large impact on people’s lives. The amount of ML solutions in medicine (research tools and commercial products) is steadily on the rise, in particular in the fields of radiology and cardiology^16,17^. Despite breakthroughs up to human-level performance^9,18–20^, ML-backed medical products are mainly used as diagnosis assistance systems^17^ leaving the final decision to medical human professionals. In particular, medical ML solutions are successfully solving the task of image segmentation^21–23^. Due to the unknown consequences of using AI for medical decision-making, more stringent regulatory requirements are of high importance to accelerate the approval process of new AI products into medical practice. Decision-making needs to be supported by reliable health data to generate consistent evidence. One of the drivers for evidence-based medicine approaches was the introduction of scientific standards in clinical practice^24^. Since then, data integrity (defined by the ALCOA-principles or ALCOA+^25^) has become an essential requirement of several guidelines, such as good clinical practice^26^, good laboratory practice^27^ or good manufacturing practice^28^. In the pharmaceutical industry, data integrity plays a similarly important role as a requirement for drug trials. While data integrity focuses on maintaining the accuracy and consistency of a dataset over its entire life cycle, data quality is concerned with the fitness of data for use.

To improve confidence in AI utilisation in general, the focus is put on the development of so-called trustworthy AI, which aims at overcoming the black box character and developing a better understanding. Several approaches and definitions for trustworthy AI have been discussed and published over the past years by researchers^29–33^, public entities^34,35^, corporations^36^, and organisations^37,38^. Depending on the area of interest, trustworthiness may include (but is far from limited to) topics such as ethics; societal and environmental well-being; security, safety, and privacy; robustness, interpretability and explainability; providing appropriate documentation for transparency and accountability^29–38^. In particular, the approach to achieve transparency through documentation has gained much attention in the form of reporting guidelines and best practices. While some initiatives cover the entire ML system and development pipeline (e.g., MINIMAR^39^, FactSheets^40^), others are concerned with documentation surrounding the model (e.g., Model Cards^41^), and still others concentrate on the documentation of datasets (e.g., Datasheets^42^, STANDING Together^43,44^, Dataset Nutrition Label^45^, Data Cards^46^, Healthsheet^47^, Data Statements for NLP^48^). These standardisation efforts are a crucial first step for developing a better understanding of ML systems as a whole and of the interdependence of its components (e.g., data and algorithm). However, these approaches cover only limited information on the content of datasets and their suitability for use in ML. Additionally, we note that reporting guidelines and best practices concerning the documentation of datasets are mostly written from the perspective of providers and creators of datasets^42,45^, with some explicitly trying to reduce information asymmetry between supplier and consumer^40^.

One of the most critical parts of an AI is the quality of its training data since it has fundamental impact on the resulting system. It lays the foundation and inherently provides limitations for the AI application. If the data used for training a model is bad, the resulting AI will be bad as well (‘garbage in, garbage out’^49^). Neural networks are prone to learning biases from training data and amplifying them at test time^50^, giving rise to a much discussed aspect of AI behaviour: fairness^51^. Many remedies have been put forward to tackle discriminating and unfair algorithm behaviour^52–54^. Yet, one of the main causes of undesirable learned patterns lies in biased training data^55,56^. Thus, data quality plays a decisive role in the creation of trustworthy AI and assessing the quality of a dataset is of utmost importance to AI developers, as well as regulators and notified bodies.

The scientific investigation of data quality was initiated roughly 30 years ago. The term data quality was famously broken down into so-called data quality dimensions by Wang and Strong in 1996^57^. These dimensions represent different characteristics of a dataset which together constitute the quality of the data. Throughout the years, general data quality frameworks have taken advantage of this approach and have produced refined lists of data quality dimensions for various fields of application and types of data. Naturally, this has produced different definitions and understandings. Within this systematic review, we transfer the existing research and knowledge about data quality to the topic of AI in medicine. In particular, we investigate the research question: Along which characteristics should data quality be evaluated when employing a dataset for trustworthy AI in medicine? The systematic comparison of previous studies on data quality combined with the perspective on modern ML enables us to develop a specialised data quality framework for medical training data: the METRIC-framework. It is intended for assessing the suitability of a fixed training dataset for a specific ML application, meaning that the model to be trained as well as the intended use case should drive the data quality evaluation. The METRIC-framework provides a comprehensive list of 15 awareness dimensions which developers of AI medical devices should be mindful of. Knowledge about the composition of medical training data with respect to the dimensions of the METRIC-framework should drastically improve comprehension of the behaviour of ML applications and lead to more trustworthy AI in medicine.

We note that data quality itself is a term used in different settings, with different meanings and varying scopes. For the purpose of this review, we focus on the actual content of a dataset instead of the surrounding technical infrastructure. We do so since the content is the part of a dataset which ML applications use to learn patterns and develop their characteristics. We thus exclude research on data quality considerations and frameworks within the topic of data governance and data management^58,59^. This concerns aspects such as data integration^60^, information quality management^61^, ETL processes in data warehouses^62^, or tools for data warehouses^63,64^ which do not affect the behavioural characteristics of AI systems. We also omit records discussing case studies of survey data quality^65,66^, as well as training strategies to cope with bad data^67–72^.

We further point out that the use of the term AI in current discussions is scientifically imprecise since discussions within the healthcare sector almost exclusively revolve around the implementation of ML approaches, in particular of DL approaches. Technically, the term AI spans a much wider range of technologies than just DL as part of the field of ML. Due to the complexity of DL applications and their proficiency in solving tasks deemed to require human intelligence, the terms are currently often used interchangeably in literature. We follow the same vocabulary here (e.g., ‘trustworthy AI’, ‘AI in medicine’) but stress the limitation of our results to ML approaches.

## Results

**Fig. 1:**
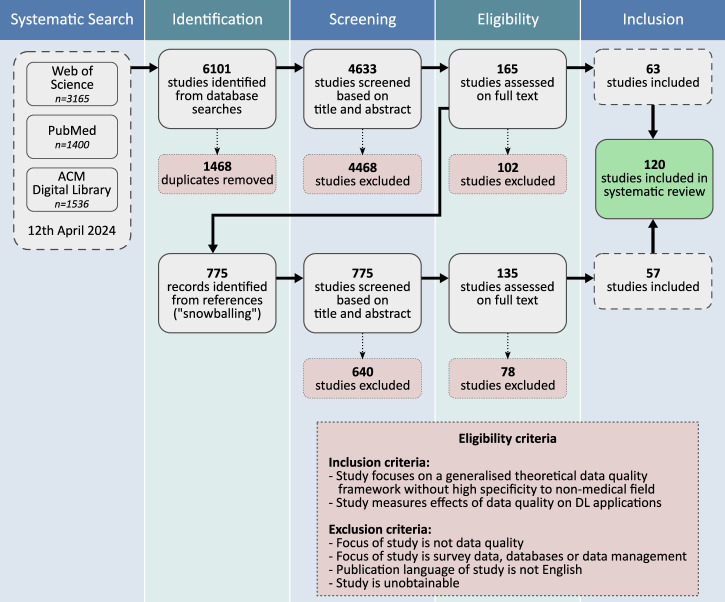
PRISMA flow diagram. The flow diagram shows the number of records identified, included and excluded at the different stages of the systematic review. The eligibility criteria for inclusion and exclusion are presented in the bottom right hand side. From a total of 5408 identified studies (4633 from database search, 775 from snowballing), the resulting literature corpus on data quality for trustworthy AI in medicine includes 120 studies.

In order to answer the research question ‘Along which characteristics should data quality be evaluated when employing a dataset for trustworthy AI in medicine?’, we conducted an unregistered systematic review following the PRISMA guidelines^73^. Our predetermined search string contains variations of the following terms: (i) data quality, (ii) framework or dimensions and (iii) machine learning (see Methods for more details and the full search string). The initial search of the databases Web of Science, PubMed and ACM Digital Library was performed on the 12th of April 2024 and yielded 4633 unique results. After title and abstract screening, adding references of the remaining records (‘snowballing’) and full text assessment, we find 120 records that match our eligibility criteria (see Methods). This represents the literature corpus that serves as a foundation for answering the research question. The full workflow is illustrated in Fig. 1.

**Fig. 2:**
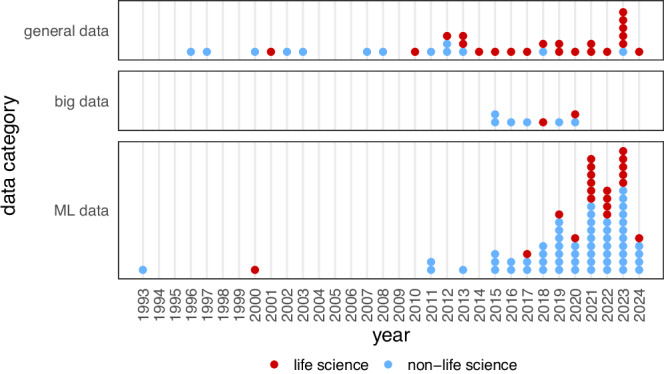
Studies included in the literature corpus sorted by publication date. The 120 studies are divided into the three categories *general data* (35), *big data* (8) and *ML data* (77), which represent major changes in the perception of data quality. The studies' affiliation to either non-life science (76) or life science (44) related topics is indicated as well.

In Fig. 2, the papers from our literature corpus are displayed according to their publication year^57,74–192^. The overarching topics contained in the corpus naturally divide the papers into three categories: *general data* (35 entries), *big data* (8 entries) and *ML data* (77 entries). This reflects the historic development of the research field of data quality during the last 30 years.

### General data quality

The field first shifted into focus with digital and automatically mass-generated data during the 1980s and 1990s causing a need for quality evaluation and control on a broad scale. While during the first 10 years landmark papers^57,74^ built the foundation for the field, the last 20 years have seen general data quality frameworks published more frequently^75–84^. The literature corpus additionally contains general data quality frameworks with high specificity to medical applications^85–105^ while frameworks with high specificity to non-medical topics^193,194^ were excluded.

The early data quality research in the 1980s and 1990s uncovered the lack of objective measures to asses data quality, which led to the introduction of task-dependent dimensions and the establishment of a data quality framework from the perspective of the data consumer^57^. Another fundamental challenge in the data quality field is the efficient data storage while maintaining quality. This was first investigated with the introduction of a data quality framework from the perspective of the data handler^74^. Both approaches to data quality proved to be useful and were unified in one framework^75^. In the following years, the frameworks were further extended^76,77^, equipped with measures^78,79^ and refined^80,81^. Moreover, it became clear that specialised fields such as the medical domain require adapted frameworks.

With the overarching question of how to improve patient care and the rise of electronic health records (EHR) in the 1990s, the need for high data quality in the medical sector increased. Accordingly, one of the first data quality frameworks in healthcare was implemented by the Canadian Institute for Health Information^85^. The first comprehensive data quality framework specifically for EHR data in the literature corpus was established by conducting a survey of quality challenges in EHR^86^. It considers, among other characteristics, accuracy, completeness and particularly timeliness. However, accuracy is hard to quantify in the medical context as even the diagnosis of experienced practitioners sometimes do not coincide. Accordingly, the notion of concordance of differing data sources was introduced^87^. Yet, the data quality frameworks for EHR could only be transferred to other types of medical data to a certain extent. Thus, data quality frameworks for particular data types such as immunisation data, public health data, multi-centre healthcare data or similar were put forward^88–95^. The various frameworks still suffered from inconsistent terminology and attempts were made to harmonise the definitions and assessment^96–103^. Particularly, Kahn et al.^97^ proposed a framework with exact definitions and recently, Declerck et al.^103^ published a ‘review of reviews’ portraying the different terminologies and attempting to map them to a reference. While these developments have advanced the understanding of data quality in the context of medical applications, frameworks for EHR frequently focus on the data quality of individual patients^86,87^, neglecting data quality aspects for the overall population. In particular, representativeness is often not a factor^86,87^ while it is a crucial property for secondary use of data in clinical studies^88^ or when reusing medical data as training data for ML applications.

### Big data quality

As the amount of data from varying sources grew, conventional databases reached their capacity and the field of big data emerged. Big data is generally concerned with handling huge unstructured data streams that need to be processed at a rapid pace, emphasising the need for extended data quality frameworks. This development is reflected by a small wave of papers published between 2015 and 2020^106–113^. For example, the weaker structure of the data encouraged the use of data quality frameworks that include the data schema as a data quality dimension^106,107^. Further, the increasing amount of data requires the computational efficiency of the surrounding database infrastructure to be a part of big data quality frameworks^108–110^. Computational efficiency is also a limiting factor when ML methods are applied to big data. While it is generally assumed that more data leads to better results, this has to be balanced with computational capabilities. Hence, a data quality framework was developed that bridges the gap between ML and big data^111^. We note that the ‘4 V’s’ (volume, velocity, veracity and variety) of big data^195^ implicitly suggest a framework for big data quality. However, the ‘4 V’s’ are in fact big data traits which can have an effect on data quality but are not considered data quality dimensions^196^. They therefore do not contribute to answering our research question and are not further discussed. This might change in the future when data from wearables or remote patient monitoring sensors become available for health management.

### ML data quality

The performance and behaviour of DL applications heavily depends on the quality of the data used during training as this is the foundation from which patterns are learned. The records of the literature corpus which discuss or empirically evaluate the effect of data quality on DL deal with a wide variety of data types and models. Many records investigate tabular data while utilising both simpler and more advanced architectures^114–132^. Recently, studies increasingly look at data quality in the context of sequential data (often time series)^133–139^, images^119,139–182^, natural language^120,121,145,182–186^ or other complex types of data^122,151,187–192^. Some papers try to estimate data quality effects on ML models by using synthetic data^122,123,189^.

Contrary to the big data and general data quality literature from our corpus, the DL papers focus on the evaluation of one or very few specific data quality dimensions without (yet) considering broader theoretical data quality frameworks. Dimensions that are predominantly investigated are those which can easily be manipulated and lend themselves to be applicable to a wide range of datasets irrespective of specific tasks. The most prominent dimension is *amount of data*^123–126,129,146–159,183–189^ which is empirically shown to benefit performance, albeit in a saturating manner. Another dominant topic is completeness to which the ML community almost exclusively refers to as *missing data*^119,125–128,133–135,182^. The effect that data errors have on the DL application is also frequently investigated. Specifically, this is done by separately looking at *perturbed features* (inputs of a NN)^128–134,136,159–168,182^ and *noisy targets* (predictions to be generated by a NN)^131–133,154–159,168–183^. Many ML settings are classification tasks which is reflected by the corpus often addressing label noise^157–159,169,182,183^. One record highlights the hefty weight that physicians’ annotations carry in medicine^158^. In order to evaluate the effect of data quality (features or targets) on ML applications, the training data is commonly manipulated. On the feature (input) side, e.g., images are distorted by adjusting contrast whereas time series sequences are disturbed by swapping elements. On the target side, e.g., correct labels are randomly replaced by false ones.

When it comes to the concrete behaviour change of the DL algorithm, most of the DL papers in the literature corpus investigate the robustness of a model, i.e. the stable behaviour of a model when facing erroneous or a limited amount of inputs. Only few records investigate generalisability^119,144,145^ or distribution shift^139,192^, a model’s capability of coping with new, unseen data. Another noteworthy exception is Ovadia et al.^145^ who additionally study predictive uncertainty.

Overall, theoretical data quality frameworks enjoy little attention by the ML community due to the novelty of the ML research field. Papers often focus on few specific data quality dimensions and tasks. Each task comes with its specific data type, necessitating different approaches to manipulate the data and measure these effects. The research dealing with the impact of manipulated data is heavily skewed towards robust behaviour in the sense of predictive performance. Other possibly affected aspects such as explainability or fairness are underrepresented and to some degree neglected which is a potential shortcoming for safety-critical applications such as medical diagnosis predictions.

### METRIC-framework for medical training data

**Fig. 3:**
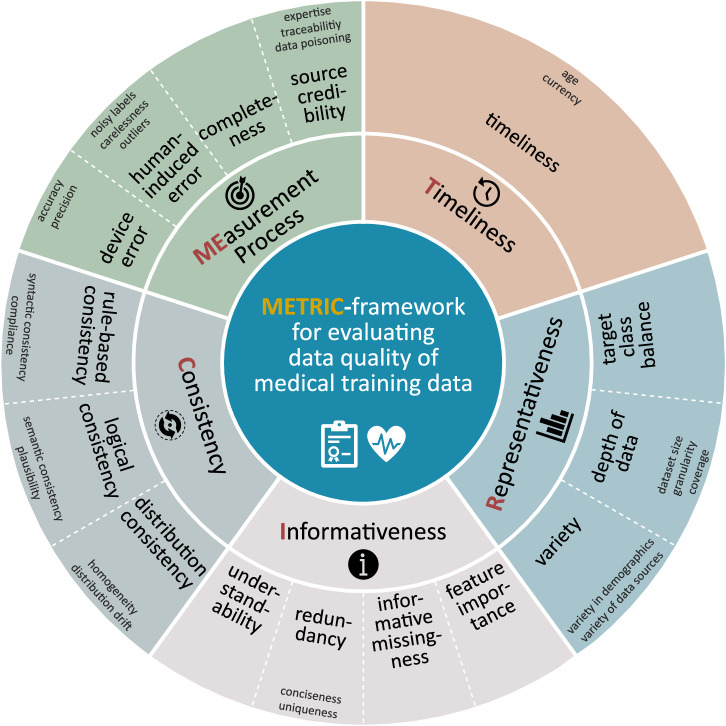
The METRIC-framework. This specialised framework for evaluating data quality of the content of medical training data includes a comprehensive set of awareness dimensions. The inner circle divides data quality into five clusters. These clusters contain a total of 15 data quality dimensions, which are shown on the outer circle. The subdimensions presented in grey on the border of the figure contribute to the superordinate dimension. Due to the shape of the graphic, we refer to it as *wheel of data quality*.

The literature corpus has shown that while similar ideas exist for the assessment of data quality across fields and applications, the idiosyncrasy of each field or application can only be captured by specialised frameworks rather than by a one-model-fits-all framework. The evaluation of data quality plays a particularly important role in the field of ML due to the fact that its behaviour is not only dependent on the algorithm choice but also strongly depends on its training data. At the same time, ML is implemented in various fields, each processing and requiring different types and qualities of data. We therefore propose a specialised data quality framework for evaluating the quality of medical training data: the METRIC-framework (Fig. 3), which is based on our literature corpus^57,74–192^. We note that the METRIC-framework is specifically not designed to assess the data quality of a dataset in vacuum. Rather, it was conceived for the situation where the purpose of the desired medical AI is known. Thus, the intention of the METRIC-framework is to assess the appropriateness of a dataset with respect to a specific use case. From now on, we refer to data quality for training (or test) data of medical ML applications only. We point out that our framework does not yet include a guideline on the assessment or measurement of data qualities but rather presents a set of awareness dimensions which play a central role in the evaluation of data quality.

While examining the literature corpus, we found that terms describing data quality appear under varying definitions, or often with no definition at all. While standardisation efforts exist for the terminology in the context of evaluating data quality^83,197,198^, they are often not employed or did not exist yet for older papers making comparisons difficult. Therefore as a first step, we extracted all mentioned data quality dimensions from the literature corpus together with their definitions (if present) and added them to a list. This yielded 461 different terms with 991 mentions across all papers. Second, we hierarchically clustered the terms with respect to their intended meaning and according to their dependencies into clusters, dimensions and subdimensions (see Methods for more details on data extraction). We thus obtained 38 relevant dimensions and subdimensions which are displayed on the outer circle of Fig. 3. In Tables 1–6, we provide a complete list of definitions for all 38 relevant dimensions and subdimensions, as well as their hierarchy, practical examples and references with respect to the literature corpus. We adopted definitions from a recent data quality glossary^197^ if they existed there and met our understanding of the dimension in the given context of medical training data. If necessary, we included definitions given by Wang et al.^57^ in a second iteration. If none of these two sources suggested an appropriate definition, we captured the meaning of the desired term on the basis of the literature corpus and thus determined its definition in the context of medical training data.

**Table 1:** Measurement Process^96,105^ cluster: definitions, examples and references

Dimension	Subdimension	Definition	Example	References
device error		The extent to which data values originating from a sensor are accurate and precise.		
	accuracy	The extent of closeness of data values to real values. (From Black and van Nederpelt^197^)	The distortion of the EEG signal due to patient movement.	^57,75–78,80,82,84–91,93,94,96,98–109,112,113,128,129,132–134,148,151,152,160–168,172^
	precision	The extent to which the error in data values spreads around zero (in statistics). (From Black and van Nederpelt^197^)	The difference between two haemoglobin level measurements from the same blood sample.	^74,75,80,90,104–106,118,131,132,152,164,182^
human-induced error		The extent to which manual inputs unintentionally influence the data in a wrong way.		^88^
	noisy labels	The extent to which labels provided by humans are accurate and precise.	The difference in the segmentation masks from two different radiologists due to different opinions or shaky hands.	^84,101,102,106,118,120,131,132,143,146,154–159,169–176,178–183,185^
	carelessness	The extent to which negligence or unintended human errors cause faulty data.	Persistent rounding of measurement readings: 120-80 systolic-diastolic heart pressure instead of actual 121-82 reading.	^57,77,78,84,85,87,90–92,96,98–102,104,109,111,112,130,131,164,182^
	outliers	The extent to which data values exist that do not match the expected distribution.	Age of a patient is greater than 150 years.	^84,98,119,131^
completeness		The extent to which data values are present. (From Black and van Nederpelt^197^)	Missing entry in dataset.	^75–79,81–84,86–92,94–99,101–107,109,111,112,117,119^^,126–128,133,134,167^^,180^^,182^
source credibility		The extent to which data values are regarded as true and believable in terms of their source. (Based on Black and van Nederpelt^197^)		^57,75–78,80,83,101,104,106–110,113,120,183^
	expertise	The extent of knowledge and experience with which the measurement was performed and the data was processed.	Diagnosis was done by a student from second semester vs. trained practitioner/ physician.	^85,90,98,104,106,112^
	traceability	The extent to which data lineage is available. (From Black and van Nederpelt^197^)	The normalisation of columns is not documented or access to raw data is not provided.	^57,74,75,80,83,105,106,110^
	data poisoning	The extent to which data values are intentionally falsified.	The cohort is selected in a way that inflates the performance.	^57,77,78,90,91,101,102,111,136,159,182^

**Table 2:** Timeliness cluster: definitions, examples and references

Dimension	Subdimension	Definition	Example	References
timeliness		The extent to which the ‘age’ and the ‘currency’ of data values are appropriate for the task at hand. (Based on Wang and Strong^57^)		^79,84,88,90,105,109,113^
	age	The extent to which the age of data values is appropriate for the task at hand. (Based on Wang and Strong^57^)	A 20-year-old dataset including measurements of patients cholesterol level is not a valid dataset for the task ‘predict the current risk of cardiovascular disease of the patient’.	^57,75–77,80,82–85,89–91,100–103,106–108,120,133^
	currency	The extent to which data values are up to date. (From Black and van Nederpelt^197^)	Updated medical knowledge leads to different diagnosis and thus label change.	^75–78,80,86–88,90,93,96,99–101,103,105,106,108,109,111^

**Table 3:** Representativeness^74,85,89,90,102–106,121^ cluster: definitions, examples and references

Dimension	Subdimension	Definition	Example	References
variety		The extent to which the dataset is diverse.		^57,113,120^
	variety in demographics	The extent to which measured subjects are diverse.	Model is supposed to be used for people of all ages, but the dataset does not contain any patient under 30.	^57,77,96,111,153,190^
	variety of data sources	The extent to which data are available from different sources. (Based on Black and van Nederpelt^197^)	Data collected from one specific device but product is supposed to run on other devices, too (in terms of different built-in hardware).	^111,128,137,143,188,191^
depth of data		The extent to which the volume of the dataset is appropriate. (Based on Wang and Strong^57^)		^57,77,78,84,104,190^
	dataset size	The extent to which the quantity of records is appropriate.	A dataset of three observations is not a solid foundation for the classification of three different classes.	^100,110,122–126,129,138,146,147,149,151–154,156,158,159,169,183–189^
	granularity	The extent to which the level of detail of the dataset and data values is appropriate.	The resolution of the CT image is too low to identify the tumour.	^74,76,83,86,88,90,92,101,102,104,107,187,189^
	coverage	The extent to which relevant subsets of the dataset satisfy the dimensions ‘variety’ and ‘target class balance’.	For the subset of a certain age class (e.g. 20-40 year olds) only healthy patient records exist although the disease exists in people of that age group in the real world.	^57,74–77,85,91,92,96,100,102,104,105,108,120,125,146^
target class balance		The extent to which the classes of a target variable have similar size.	The dataset includes only one record of a patient with the rare disease that the model is supposed to predict.	^115,116,122,138,140–142,144,150,155,159^

**Table 4:** Informativeness^80^ cluster: definitions, examples and references

Dimension	Subdimension	Definition	Example	References
understandability		The extent to which data values are clear without ambiguity and easily comprehended. (From Wang and Strong^57^)	Physician’s diagnosis (comment in natural language) is written ambiguously such that no explicit label/ICD code can be assigned.	^57,74,76–78,80,83,85,88–90,98,100,103,104,106,108,113^
redundancy		The extent to which logically identical data are stored more than once. (From Black and van Nederpelt^197^)		^84,103,110,150^
	conciseness	The extent to which data values, attributes and records are compactly represented. (Based on Wang and Strong^57^)	One column for each possible diagnosis: Column ‘Normal’ (yes/no), ‘Myocardial Infarction’ (yes/no) instead of a single column ‘diagnosis’ (‘Normal’/‘Myocardial Infarction’).	^57,76–78,80,83,84,91,95,104,106,111^
	uniqueness	The extent to which records occur only once in a data file. (From Black and van Nederpelt^197^)	Same record occurs more than once.	^74–76,76,77,82,84,93–95,97,98,100,102,103,105,106,111,114,116,118–120,128,155^
informative missingness		The extent to which missing data values provide useful information.	If ‘cholesterol level’ is empty in EHR patient record, then it is likely, that the doctor never felt the need to measure it. This could indicate, that the risk for a cardiovascular disease is low.	^96,98,99,105,111,119,126,135,182^
feature importance		The extent to which the attributes are beneficial and provide advantages from their use. (Based on ‘usefulness’ from Wang and Strong^57^)	The feature ‘insurance company’ carries less information relevant for the prediction of a stroke than the feature ‘blood pressure’.	^57,74,76–78,80,85,88,90,91,96,100–109,111,112,120,143,149,150,153,187^

**Table 5:** Consistency^76,82–84,89,90,98,101,103–106,120^ cluster: definitions, examples and references

Dimension	Subdimension	Definition	Example	References
rule-based consistency		The extent to which data values comply with a rule. (‘Validity’ from Black and van Nederpelt^197^)		
	syntactic consistency	The extent to which data values comply to a dictionary and are always presented in the same format. (Based on Wang and Strong^57^)	A mixture of ‘m’ and ‘male’ violates syntactic consistency.	^57,74,75,77–84,90–98,100–106,108,109,111–113,116,119,120,127,133^
	compliance	The extent to which the dataset and data values are in accordance with laws, regulations or standards. (From Black and van Nederpelt^197^)	All entries of the dataset follow the ICD-10-CM coding.	^75,80,81,83,85,90,96,98,102,107,111^
logical consistency		The extent to which data values are plausible and records are semantically consistent.		^106^
	semantic consistency	The extent to which records are free of contradictions.	‘Abnormal radiologic findings on diagnostic imaging of left kidney’, but the left kidney was donated before this diagnosis was made.	^74,75,77,80,81,83–87,90–96,98,99,101–108,110–113,117,120,128^
	plausibility	The extent to which data values match knowledge of the real world. (From Black and van Nederpelt^197^)	Patient is 5 years old and 180cm tall.	^57,75,77,78,87,93,95,97,99,102,103,105,106,112,120,128^
distribution consistency		The extent to which distributions are stable.		^139,177^
	homogeneity	The extent to which distributions are stable among relevant subsets.	Data originates from two devices with different underlying calibrations.	^74,84,102,144,171,186^
	distribution drift	The extent to which new data matches the distribution of existing data.	The median age of the population shifts over the decades.	^75,77,79,85,93,100,102,145,192^

**Table 6:** Data Management cluster: definitions and references

Dimension	Definition	References
documentation	Metadata about the dataset.	^74,75,77,85,89,96,102,104,106–108,111^
security	A characteristic that ensures that unauthorised changes to the dataset are prohibited.	^57,77,78,80,89,90,103,104,106,109,113^
privacy	A characteristic that ensures that the dataset is recorded and stored in a way that protects personal data.	^75,83,89,90,104,109–111,165,167^
FAIR principles	The FAIR Principles^199^ require a dataset to be findable, accessible, interoperable and reusable.	^57,74–78,80,83,85,88,90,100–104,106,108–113^

**Fig. 4:**
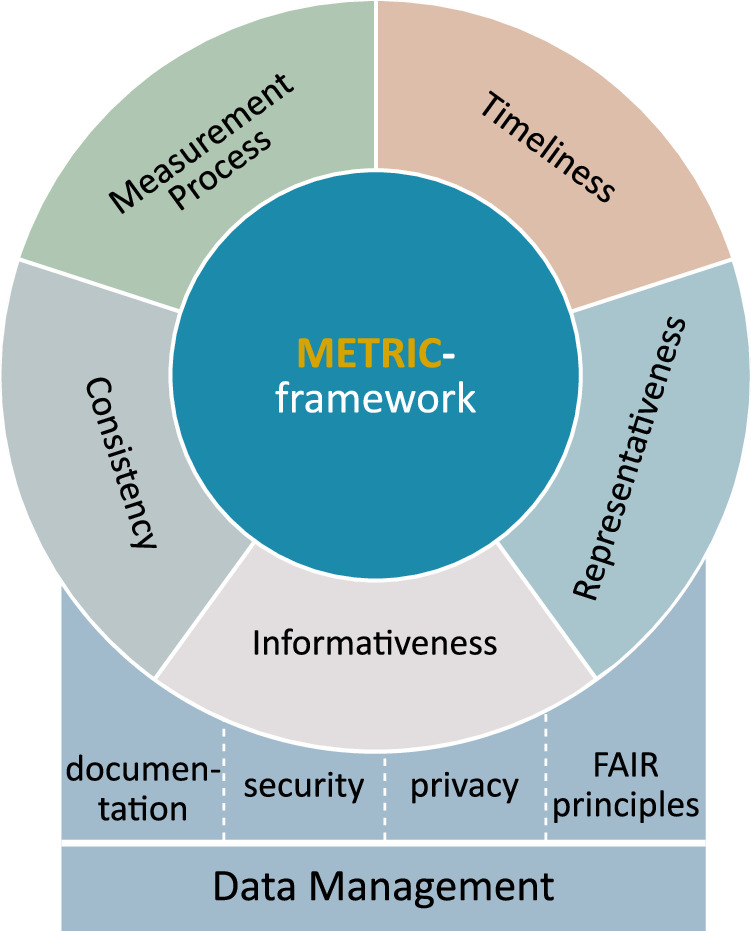
METRIC-Framework in relation to data management. The cluster *data management* is concerned with the effective usage of the dataset. It includes basic requirements for the dataset but does not address data quality issues regarding its content. Therefore, it can be seen as a prerequisite for assessment using the METRIC-framework. Figuratively speaking, the *data management* cluster serves as a stable foundation for the wheel of data quality.

The METRIC-framework encompasses three levels of details: *clusters* which pool similar dimensions; *dimensions* which are individual characteristics of data quality; and *subdimensions* which split larger dimensions into more detailed attributes (compare Fig. 3 from inside to outside). Besides the terms contained in the METRIC-framework, we found several frequently mentioned dataset properties which we, for our purpose, want to separate from the METRIC-framework. We summarise these additional properties under a separate cluster called *data management* (Fig. 4). The attributes included in this cluster ensure that a dataset is well-documented, legally and effectively usable. In particular, it includes the properties *documentation*, *security* and *privacy*, as well as the well-established *FAIR-Principles*^199^. Appropriate *documentation* of datasets is the topic of multiple initiatives^42–47^ that give guidance for the data creator and handler. The METRIC-framework on the other hand is targeted towards AI developers. It evaluates the suitability of the content of the data for a specific ML task, which is greatly facilitated by appropriate documentation but does not depend on it. Similarly, the *FAIR-principles*^199^, requiring data to be findable, accessible, interoperable and reusable, are vital for evaluating datasets for general purpose but are not included in the METRIC-framework since the question of fit for a specific purpose can only be asked when a dataset is already successfully obtained. *Security* is another important aspect of data management: Who can access and edit the data? Can it be manipulated? Again, such questions concern the handling of the data, not the evaluation of its content. Finally, *privacy* (data privacy and patient privacy) is a delicate and heavily discussed topic in the context of healthcare. However, we separate these issues from the METRIC-framework since they concern data collection, creation and handling. We note that aspects such as anonymisation or pseudonymisation may impact the quality of the content of a dataset by, e.g., removing information^167^. However, the METRIC-framework is designed to evaluate the resulting dataset with respect to its usefulness for a specific task, not the quality of the modifications. Hence, while these properties play a central role in the creation, handling, management and obtainment of data, the METRIC-framework is targeted at the content of a dataset since that is the part the ML algorithm learns from. Therefore, we see the *data management* cluster as a prerequisite for data quality assessment by the METRIC-framework which itself divides the concept of data quality for the content of a dataset into five clusters: *measurement process*, *timeliness*, *representativeness*, *informativeness*, *consistency*. A summary of the characteristics and key aspects of all five clusters is given in Table 7.

**Table 7:** Key characteristics of each of the five clusters of the METRIC-framework

Cluster	Description
Measurement process	Concerned with technical or human influences that affect the data acquisition process. Includes ‘device errors’ leading to poor accuracy and precision, ‘human-induced errors’ such as noisy labels, ‘completeness’ counting the number of missing values, and ‘source credibility’ estimating the reliability of the data.
Timeliness	Concerned with changes in time and whether they are appropriately reflected in the dataset. Includes ‘age’ concerned with the relation between creation date and the date of usage, as well as ‘currency’ representing the updatedness of the data.
Representativeness	Concerned with the appropriate and comprehensive representation of the targeted population in the dataset. Includes ‘variety’ indicating whether a sufficiently broad range of demographics and data sources is present, ‘depth of data’ investigating whether the overall amount of data as well as coverage of subpopulations is sufficient, and ‘target class balance’ judging whether classes of the target variable are appropriately sized for use in ML.
Informativeness	Concerned with how well the data conveys the information it describes. Includes ‘understandability’ indicating whether data is easily comprehended and without ambiguity, ‘redundancy’ investigating whether information is provided more than once, ‘informative missingness’ quantifying whether missing values carry additional information, and ‘feature importance’ estimating the value features add for the application.
Consistency	Concerned with the consistency of presentation and composition of the dataset. Includes ‘rule-based consistency’ investigating whether data is presented in a consistent format that follows internal and external rules, ‘logical consistency’ judging whether data is logically sound and without contradictions, and ‘distribution consistency’ considering whether different subpopulations have similar statistical properties.

### Measurement process

The cluster *measurement process* captures factors that influence uncertainty during the data acquisition process. Two of the dimensions within this cluster differentiate between technical errors originating from devices during measurement (see *device error*) and errors induced by humans during, e.g., data handling, feature selection or data labelling (see *human-induced error*). For the dimension *device error*, we distinguish between the subdimension *accuracy*, the systematic deviation from the ground truth (also called bias), and the subdimension *precision*, the variance of the data around a mean value (also called noise). In practice, a ground truth for medical data is most often not attainable, making *accuracy* evaluation impossible. In that case, the level and structure of noise in the training data should be compared to the expected noise in the data after AI deployment. If the training data only contains low noise but the AI is utilised in clinical practice on data with much higher noise levels, the performance of the AI application might not be sufficient since the model did not face suitable error characteristics during training. Therefore, lower noise data is not necessarily better and adding noise to the training data might in some instances even improve performance^200–202^. The errors belonging to the dimension *human-induced errors* are of a fundamentally different nature and need to be treated accordingly. This type of error includes human *carelessness* and *outliers* in the dataset due to (unintentional) human mistakes. The final subdimension, *noisy labels*, is one of the most relevant topics in current ML research^157,159,169,182^. Since in the medical domain, supervised learning paradigms are prevalent, proper feature selection and reliable labelling are indispensable. However, human decision making can be highly irrational and subjective, especially in the medical context^203–205^, representing one of various sources of labelling noise^206^. Among expert annotators there is often considerable variability^206,207^. Even in the most common (non-medical) datasets of ML (e.g., MNIST^208^, CIFAR-100^209^, Fashion-MNIST^210^) there is a significant percentage of wrong labels^211,212^. In contrast to the precision of instruments, noise in human judgements is demanding to be assessed through so-called noise audits to identify different factors, like pattern noise and occasion noise in the medical decision process^213^. Such intra- and inter-observer variability has always been a highly important topic in many medical disciplines, e.g., in radiology where guidelines, training and consensus reading approaches are used to reduce noise^214^.

Another issue that frequently occurs in the data acquisition process and which plays an important role in ML is the absence of data values with unknown reason. We follow the ML vocabulary by capturing this quality issue with the dimension *completeness*, while noting that outside of ML contexts, this term is commonly used to describe representativeness, coverage or variety in other contexts. Most prominently, Wang et al.^57^ define completeness as ‘breadth, depth, and scope of information’. This definition has been picked up by other researchers, as well^100,106,126^. In ML, however, *completeness* is usually measured by the ratio of missing to total values. Apart from the mostly quantitative dimensions within the cluster, the dimension *source credibility* is concerned with mostly qualitative characteristics. On the one hand, it includes the question whether or not the measured data can be trusted based on the *expertise* of people involved in data measurement, processing and handling. On the other hand, the subdimension *traceability* evaluates whether changes from original data to its current state are documented. Being aware of modifications such as the exclusion of outliers, automated image processing in medical imaging or data normalisation and their utilised algorithms are necessary for understanding the composition of the data. Finally, the subdimension *data poisoning* considers whether the data was intentionally corrupted (e.g., adversarial attacks) to cause distorted outcomes. The entire cluster *measurement process* is crucial for data quality evaluation in the medical field since errors may propagate through the ML model and lead to false diagnosis or treatment of patients.

We note that special consideration has to be given to the field of medical imaging within the *measurement process* cluster due to the fact that many imaging devices are not classical measurement devices. For instance, in current radiological practice, decisions are still based mainly on visual inspection of images and rating of diseases and therapy effects are often done in qualitative terms such as ‘enlarged’, ‘smaller’ or ‘enhanced’. This places a lot of importance on the qualitative subdimensions *source credibility* and *expertise*, with respect to quality assessment in such use cases. However, over the last two decades significant efforts have been made to establish quantitative imaging biomarkers to transform scanners more into measurement devices to quantify biophysical parameters, like flow, perfusion, diffusion or elasticity. Such quantitative imaging approaches reduce the operator dependency and enable more quantitative evaluation in the dimension *device error*. Worldwide alliances such as Quantitative Imaging Biomarkers Alliance (QIBA) launched in 2007 by the Radiology Society North America^215^ and now replaced by the Quantitative Imaging Committee (QUIC), the Quantitative Imaging Network (QIN) of the National Cancer Institute in the US^216^ or the European Imaging Biomarkers Alliance (EIBALL) by European Society of Radiology^217^ are committed to make this transformation.

### Timeliness

Since medical knowledge and understanding is subject to constant development, it is important to investigate the cluster *timeliness* which indicates whether the point in time at which the dataset is used in relation to the point in time at which it was created and updated is appropriate for the task at hand. Indications for diagnoses based on medical data may have changed since a dataset was created and labelled, and changes in coding systems (such as the transition from ICD-9 to ICD-10 or ICD-9-CM to ICD-10-CM) may affect mortality and injury statistics^218,219^. The *age* of the data dictates whether such investigations are necessary. In such cases, the labels or standards utilised would then have to be appropriately updated to satisfy the subdimension *currency*. Furthermore, knowledge about the subdimension *age* might provide information about precision and accuracy of the measurement as it gives insight into the technology used during data acquisition.

### Representativeness

Another central cluster, especially for medical applications, is *representativeness*. Its dimensions are concerned with the extent to which the dataset represents the targeted population (such as patients) for which the application is intended. Whether the population of the dataset covers a sufficient range in terms of age, sex, race or other background information is the topic of the subdimension *variety in demographics* contained within the dimension *variety*. This dimension also contains the subdimension *variety of data sources* concerned with questions such as: Does the data originate from a single site? Were the measurements done with devices from the same or different manufacturers? Appropriately investigating such questions can provide a strong indication for the applicability and generalisability of the ML application in different environments^220–223^. The dimension *depth of data* is one of the main topics of the ML papers in our literature corpus. Apart from the subdimension *dataset size* already discussed in the previous section, this dimension also includes the subdimension *granularity*, which considers whether the level of detail (e.g., the resolution of image data) is sufficient for the application, as well as the subdimension *coverage*, which investigates whether sub-populations (e.g., specific age groups) are still diverse by themselves (e.g., still contain all possible diagnoses in case of classification applications). Finally, the highly-discussed dimension *target class balance* pays tribute to the technical requirements of ML^140,141,144,150,159^. An algorithm must learn patterns for specific classes from the training data. However, strong imbalances in the class ratio could be caused by, e.g., rare diseases. In order to still be able to properly learn corresponding patterns it may be helpful to deliberately overrepresent rare classes in the dataset instead of matching their real world distribution^224,225^.

### Informativeness

The cluster *informativeness* considers the connection between the data and the information it provides and whether the data does so in a clear, compact and beneficial way. First of all, the *understandability* of the data considers whether the information of the data is easily comprehended. Second, the dimension *redundancy* investigates whether such information is concisely communicated (see subdimension *conciseness*) or whether redundant information is present such as duplicate records (see subdimension *uniqueness*). The dimension *informative missingness* answers the question whether the patterns of missing values provide additional information. Che et al.^135^ find an informative pattern in the case of the MIMIC-III critical care dataset^226^ which displays a correlation between missing rates of variables and ICD9-diagnosis labels. Missingness patterns are categorised by the literature into either *not missing at random* (NMAR), *missing at random* (MAR) or *missing completely at random* (MCAR)^227,228^. Finally, *feature importance* is concerned with the overall relevance of the features for the task at hand and moreover with the value each feature provides for the performance of a ML application since the quantity of data has to be balanced with computational capability. Valuable features might in many cases be as important as dataset size^229^, which is a frequently discussed topic in the data-centric AI community^230^.

### Consistency

The dimensions belonging to the cluster *consistency* illuminate the topic of consistent data presentation from three perspectives. *Rule-based consistency* summarises subdimensions concerned with format (*syntactic consistency*), which includes the fundamental and well-discussed topic of data schema^106^, and the conformity to standards and laws (*compliance*). These subdimensions ensure that the dataset is easily processable on the one hand and comparable and legally correct on the other. *Logical consistency* evaluates whether or not the content of the dataset is free of contradictions, both within the dataset (e.g., a patient without kidneys that is diagnosed with kidney stones) and in relationship to real world knowledge (e.g., a 200-year-old patient). The last dimension of the cluster, *distribution consistency*, concerns the distributions and their statistical properties of relevant subsets of the total dataset. While the subdimension *homogeneity* evaluates whether subsets have similar or different statistical properties at the same point in time (e.g., can data from different hospitals be identified by statistics?), the subdimension *distribution drift* deals with varying distributions at different time points. This subdimension can be neglected if the dataset is not continuously changing over time, but distribution drift is sometimes unconsciously discarded due to a lack of model surveillance. Therefore, it is a prominent research topic^145^ and the unconsciousness furthermore underlines the importance of *distribution drift* for medical applications^93^.

## Discussion

The METRIC-framework (Fig. 3) represents a comprehensive system of data quality dimensions for evaluating the content of medical training data with respect to an intended ML task. We stress again that these dimensions should for now be regarded as awareness dimensions. They provide a guideline along which developers should familiarise themselves with their data. Knowledge about these characteristics is helpful for recognising the reason for the behaviour of an AI system. Understanding this connection enables developers to improve data acquisition and selection which may help in reducing biases, increasing robustness, facilitating interpretability and thus has the potential to drastically improve the AI’s trustworthiness.

With training data being the basis for almost all medical AI applications, the assessment of its quality gains more and more attention. However, we note that providing a division of the term data quality into data quality dimensions is only the first step on the way to overall data quality assessment. The next step will be to equip each data quality dimension with quantitative or qualitative measures to describe their state. The result of this measure then has to be evaluated with respect to the question: Is the state of the dimension appropriate for the desired AI algorithm and its application? These three steps (choosing a measure, obtaining a result, evaluating its appropriateness for the desired task) can be applied to each dimension and subdimension. Appropriately combining the individual outcomes can potentially serve as a basis for a measure of the overall data quality in future work.

So far the dimensions in the METRIC-framework are not ranked in any way. However, it is clear that some of them are more important than others. Therefore, some dimensions deserve more attention in the assessment process or might even be a criterion for exclusion of a dataset for a certain task. These dimensions should be among the first to be assessed in practice. On the other hand, some dimensions are much more difficult to measure and evaluate than others. This can be due to their qualitative nature, the complexity of the statistical measure, the degree of use-case dependence or the expert knowledge that is needed for the assessment, to name a few. These considerations are of central interest for the development of a complete data quality assessment and examination process.

**Fig. 5:**
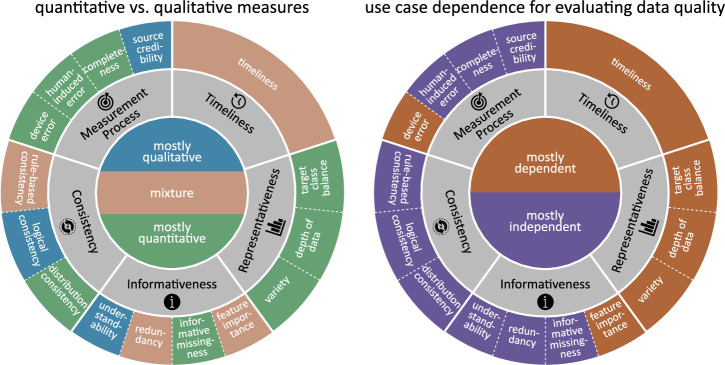
Categorisation of the METRIC-framework. Categorisation of dimensions along the properties *quantitative vs. qualitative measure* (left) and *use case dependence for evaluating data quality* (right). The affiliation to a category is colour-coded. The colour scale is presented in the inner circle.

In Fig. 5, we provide insights that should be taken into consideration when practically assessing data quality. We classify each of the 15 awareness dimensions along two different properties. On the one hand, we estimate whether a dimension requires mostly *quantitative* or *qualitative* measures. We observe that about half of the dimensions require mostly quantitative measures while a fifth necessitate more manual inspection by qualitative measures (see left-hand side of Fig. 5). Being able to choose quantitative measures typically implies more objectivity and enables automation, two desirable properties for quality assessment. Dimensions categorised as mostly qualitatively measurable or requiring a mixture of quantitative and qualitative input will typically require specific domain knowledge from the medical field. Such domain knowledge can be difficult to obtain and expensive.

On the other hand, we consider whether the state of a dimension or the evaluation of its appropriateness level is *use case dependent* (see right-hand side of Fig. 5). This is of interest to developers as use case dependent dimensions require not only additional knowledge, work and time during quality assessment but also during quality improvement of data. Our findings suggest a division of the wheel of data quality after categorising all 15 dimensions. The clusters *representativeness* and *timeliness* as well as the dimensions *device error* and *feature importance* belong to the group of use case dependent dimensions. Whether a dataset is representative of the targeted population can only be evaluated with knowledge of the use case. Similarly, the importance of features changes between applications. Whether the age and currency of the data (see dimension *timeliness*) are appropriate can also differ depending on the task. For instance, the coding standard the data should conform to depends on the application. The newest standards are not necessarily the best if in practice these standards are not implemented (see section on *Timeliness*). Similarly, reducing noise levels in the data is not necessarily better for all applications. It rather depends on the expected noise levels of the application (see section on *Measurement process* for more detail).

For an overall assessment of the quality of the dataset, we estimate that on average the dimensions of the *representativeness* cluster together with the dimensions *feature importance*, *distribution consistency* and *human-induced error* are crucial factors. Ignoring a single one of these dimensions potentially has proportionally larger effects on the AI application than other dimensions. This might also depend on the type of ML problem. Actual quantification of the effect of data quality dimensions on ML applications is part of ongoing and future research. Nevertheless, we for now recommend prioritising these six dimensions if it is possible to dedicate time to evaluating or improving a dataset. With the exception of the dimension *feature importance*, all of the crucial dimensions are simultaneously measured mostly quantitatively making them primary candidates for software tools designed for improving the quality of datasets.

The importance of data quality for medical ML products is undisputed and gaining more and more attention with on-going discussions about fairness and trustworthiness. Parts of future regulation and certification guidelines will not only include ML algorithms but likely also require evaluating the quality of datasets used for their training and testing. Such inclusion of data quality in regulation requires systematic assessment of medical datasets. The METRIC-framework may serve as a base for such a systematic assessment of training datasets, for establishing reference datasets, and for designing test datasets during the approval of medical ML products. This has the potential to accelerate the process of bringing new ML products into medical practice.

## Methods

### Literature review

In order to answer the research question ‘Along which characteristics should data quality be evaluated when employing a dataset for trustworthy AI in medicine?’, we conducted a systematic review following the PRISMA guidelines^73^. The goal of such a review is to objectively collect the knowledge of a chosen research area by summarising, condensing and expanding the ideas to further its progress. PRISMA reviews commonly follow four main steps: (i) Searching suitable databases with carefully formulated search strings and extracting matching papers; (ii) screening titles and abstracts to include or exclude papers based on predetermined criteria; (iii) extending the literature list by screening titles and abstracts of all referenced papers from the included papers (called ‘snowballing’); (iv) screening the full text of all still included papers with respect to the eligibility criteria to build the final literature corpus.

### Search strategy

Our research question aims at combining the knowledge from the field of general data quality frameworks with insights about the effects that the quality of training data has on ML applications in medicine. This should ultimately lead to a novel framework for data quality in the context of medical training data. Therefore, we built a search string that on the one hand targeted papers about data quality frameworks by combining variations of ‘data quality’ with variations of the terms ‘framework’ and ‘dimensions’. On the other hand, we attempted to collect papers about the connection between the quality of training data and the behaviour of a DL application by again combining variations of the word ‘data quality’ but this time with variations of ‘machine learning’, including ‘artificial intelligence’ and ‘deep learning’ (see Search query). We then performed the database search on one general and two thematically suitable online databases: Web of Science, PubMed and ACM Digital Library. We are aware that the choice of databases skews, to some degree, all interpretations which, to some extent, is mitigated by snowballing. All retrieved results were concatenated and duplicates removed, yielding 4633 records.

### Search query

**Fig. 6:**
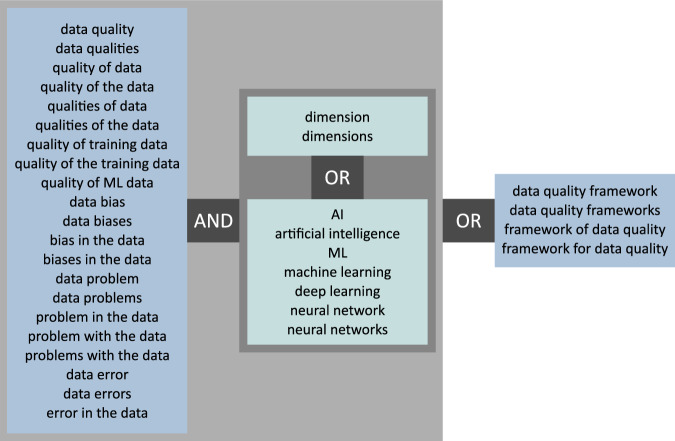
Search string visualisation. Visualisation of the keywords and logical connections that formed the search string. Each box can be translated to parantheses in the search string. Keywords inside each box are connected with each other by a logical *OR*.

The following search string in pseudo-code (visualised in Fig. 6) was executed on the 12th of April 2024 on Web of Science, PubMed and ACM Digital Library:

(("data quality" OR "data-quality"  OR "data qualities" OR "quality of data"  OR "quality of the data" OR "qualities of data"  OR "qualities of the data" OR "quality of training data"  OR "quality of the training data" OR "quality of ML data"  OR "data bias" OR "data biases"  OR "bias in the data" OR "biases in the data"  OR "data problem" OR "data problems"  OR "problem in the data" OR "problem with the data"  OR "problems with the data" OR "data error"  OR "data errors" OR "error in the data" ) AND ("dimension" OR "dimensions"  OR "AI" OR "artificial intelligence"  OR "ML" OR "machine learning"  OR "deep learning"  OR "neural network" OR "neural networks" ))OR("data quality framework" OR "data quality frameworks" OR "framework of data quality" OR "framework for data quality") The chosen databases supported exact (instead of fuzzy) searches, expressed by quotation marks around keywords. The search was applied to the title and abstract fields of all records of the databases.

### Eligibility criteria

In Table 8, our chosen eligibility criteria that were applied to the various screening steps are listed. Papers were included if they either provided broad-scale data quality frameworks with general purpose or with specificity to a medical application, or if they discussed or quantified the effects of at least one training data quality dimension on DL behaviour. In contrast, papers were excluded if they (i) either discussed frameworks with specificity to non-medical fields or (ii) only considered single or few data quality dimensions without reference to ML or (iii) focused on the quality of data management and surveys. No limits were imposed with respect to publication date or publisher source (i.e. peer-reviewed or not), while non-English records and inaccessible records were omitted.

**Table 8:** Eligibility criteria applied to the screening and full-text assessment processes

Criterion	Reasoning
*Inclusion*	
Study focuses on a generalised theoretical data quality framework without high specificity to non-medical field	Incorporate systematic work on data quality aspects
Study measures effects of data quality on DL applications	Get an insight into what dimensions are being investigated by the literature in the context of ML
*Exclusion*	
Focus of study is not data quality	Not related to research question
Focus of study is survey data, databases or data management	Out of scope w.r.t. our research question; too specific topics without accounting for task-specific content
Publication language of study is not English	/
Study is unobtainable	/

We note that in order to be as precise and logical as possible during the practical screening and eligibility checks, we implemented the following eligibility criteria: (I1) Inclusion: No exclusion criteria apply; (I2) Inclusion: Study measures effect of data on DL; (E1) Exclusion: Focus of study is not data quality; (E2) Exclusion: Focus of study is not on general theoretical data quality framework; (E3) Exclusion: Study has high specificity to non-medical field; (E4): Exclusion: Focus of study is quality of data management or surveys. The logic we applied during screening and eligibility check is: If any exclusion criteria applies, the study is excluded, *unless* an inclusion criteria applies at the same time.

### Literature review process

Titles and abstracts from the records of the database search were screened with respect to the eligibility criteria. This was done by two authors independently to mitigate biases. In case of disagreement, consensus was achieved by discussion. If necessary, a third author was consulted to arrive at the final decision. This step reduced the number of records to 165. The snowballing step expands the scope of the literature corpus to make it more independent of the initially chosen databases and search string which is important to reduce bias. For the process of snowballing, we considered all references from the so far 165 included papers which resulted in adding 775 records to the literature list. Analogously, title and abstract screening was performed on these new entries with the same criteria and workflow as before, leaving 135 additional papers from snowballing. As a final step, all 300 remaining papers were evaluated on the full text with respect to the eligibility criteria. In the end, 120 entries passed all screening steps. For each retrieved record, the decision whether to include or exclude was documented along with the corresponding eligibility criterion. Each record which had passed the screening was eligible for extracting data quality terms.

### Data extraction strategy

In order to introduce a comprehensive data quality framework, the 120 selected records were each read by two authors and all terms that were deemed relevant to describe data quality were extracted. See Table 9 for details on extracted vocabulary from each record. We discarded terms if (i) their scope is limited to a specialised data source and not transferable to a general framework, (ii) the term refers to the quality of database infrastructure or (iii) no definition was given and it was impossible to grasp the intended meaning from the context. The accepted terms were copied into an Excel sheet, which served as a starting template for the METRIC-framework. We clustered related concepts into groups according to the terms’ definition or intended meaning. From these small and detailed groups we formed the so-called subdimensions, ensuring that each subdimension is mentioned by at least three references in the literature corpus, otherwise the level of detail was deemed too great leading to further grouping.

**Table 9:** List of all publications in the literature corpus with the originally mentioned data quality dimensions mapped to the corresponding dimension or subdimension of the METRIC-framework

Datatype	Year	Author	Field	Measurement Process	Timeliness	Representativeness	Informativeness	Consistency
general data	1996	Wang, Strong^57^	non-life science	accuracy (accuracy), carelessness (objectivity), source credibility (reputation), traceability (traceability), data poisoning (objectivity)	age (timeliness)	variety (variety of data and data sources), variety in demographics (completeness), depth of data (amount of data), coverage (completeness)	understandability (interpretability, ease of understanding), conciseness (conciseness), feature importance (value-added (usefulness), task relevance, completeness)	syntactic consistency (representational consistency), plausibility (believability)
general data	1997	Redman^74^	non-life science	precision (precision), traceability (identify ability)		Representativeness (naturalness), granularity (granularity, format precision), coverage (comprehensiveness)	understandability (interpretability, format appropriateness), uniqueness (minimum unnecessary redundancy), feature importance (essentialness, task relevance)	syntactic consistency (structural consistency, representation consistency), semantic consistency (semantic consistency), homogeneity (homogeneity, robustness)
general data	2000	Yoon, Aiken, Guimaraes^76^	non-life science	accuracy (accuracy, correctness), completeness (completeness), source credibility (reliability)	age (timeliness), currency (currency)	granularity (precision, attribute granularity, detail), coverage (comprehensiveness)	understandability (interpretability, naturalness, clarity, unambiguousness, robustness, presentation appropriateness, media, order), conciseness (conciseness, essentialness), uniqueness (minimally redundant, occurence identifiability), feature importance (relevance)	Consistency (Consistency)
general data	2001	Richards, Seko^85^	life science	accuracy (accuracy), carelessness (simple response bias, item (partial) non-response, correlated response bias), expertise (processing)	age (timeliness)	Representativeness (relevance), coverage (over-coverage, under-coverage, comprehensiveness)	understandability (interpretability, adaptability), feature importance (reliability)	compliance (standardisation), semantic consistency (comparability), distribution drift (historical comparability)
general data	2002	Pipino, Lee, Wang^78^	non-life science	accuracy (free-of-error), carelessness (objectivity), completeness (completeness), source credibility (reputation), data poisoning (objectivity)	currency (timeliness)	depth of data (amount of data)	understandability (understandability, interpretability), conciseness (conciseness), feature importance (value-added (usefulness), task relevance)	syntactic consistency (representational consistency), plausibility (believability)
general data	2003	Kim, Choi, Hong, Kim, Lee^81^	non-life science	completeness (missing data)				syntactic consistency (integrity, ambiguous data, Different representations of compound data), compliance (non-standard conforming data), semantic consistency (Inconsistency across multiple tables/file, Different data for the same entity)
general data	2007	Stvilia, Gasser, Twidale, Smith^80^	non-life science	accuracy (accuracy), precision (precision), source credibility (authority), traceability (verify ability)	age (currency), currency (volatility)		Informativeness (Informativeness), understandability (complexity), conciseness (cohesiveness), feature importance (relevance)	syntactic consistency (intrinsic accuracy/validity, intrinsic structural consistency, intrinsic naturalness, intrinsic semantic consistency), compliance (relational semantic consistency, relational structural consistency), semantic consistency (accuracy/validity, relational accuracy)
general data	2008	International Organization for Standardization and International Electrotechnical Commission^83^	non-life science	completeness (completeness), source credibility (source credibility, authenticity), traceability (traceability)	age (currency)	granularity (precision (no. of digits))	understandability (understandability), conciseness (efficiency)	Consistency (Consistency), syntactic consistency (syntactic accuracy), compliance (compliance), semantic consistency (semantic accuracy)
general data	2010	Chan, Fowles, Weiner^86^	life science	accuracy (accuracy), completeness (completeness)	currency (timeliness)	granularity (granularity, clinical specificity)		semantic consistency (comparability)
general data	2011	Loshin^75^	non-life science	accuracy (value accuracy), precision (precision), completeness (Null validation), source credibility (authoritative sources), traceability (originating data source)	age (age), currency (correction/update promulgation)	coverage (coverage, population density optionality)	uniqueness (Entity uniqueness)	syntactic consistency (syntactic consistency, Name ambiguity), compliance (standards and policies), semantic consistency (semantic consistency, multi-value consistency), plausibility (temporal consistency, reasonableness), distribution drift (temporal reasonability)
general data	2012	Nahm^88^	life science	accuracy (accuracy), human-induced error (human-induced error), completeness (completeness)	timeliness (timeliness), currency (currency, volatility)	granularity (granularity, precision (no. of digits))	understandability (readability/legibility), feature importance (relevance)	
general data	2012	Sidi^77^	non-life science	accuracy (accuracy, free-of-error, reliability), carelessness (objectivity), completeness (completeness), source credibility (reputation), data poisoning (objectivity)	age (freshness), currency (freshness)	variety in demographics (completeness), depth of data (amount of data), coverage (coverage, completeness)	understandability (understandability, interpretability), conciseness (conciseness), uniqueness (duplication), feature importance (value-added (usefulness), Usability, completeness, relevance)	syntactic consistency (representational consistency, consistency, data integrity fundamentals), semantic consistency (consistency, consistency and synchronisation), plausibility (believability), distribution drift (data decay)
general data	2012	Sebastian-Coleman^79^	non-life science	completeness (completeness)	timeliness (timeliness)			syntactic consistency (validity), distribution drift ((temporal) consistency)
general data	2013	DAMA UK Working Group on Quality Dimensions^82^	non-life science	accuracy (accuracy), completeness (completeness)	age (timeliness)		uniqueness (uniqueness)	Consistency (Consistency), syntactic consistency (validity, consistency)
general data	2013	Almutiry, Wills, Alwabel, Crowder, Walters^89^	life science	accuracy (accuracy), completeness (completeness)	age (timeliness)	Representativeness (relevance)	understandability (usability, interpretability)	Consistency (Consistency)
general data	2013	Weiskopf, Weng^87^	life science	accuracy (correctness), carelessness (correctness), completeness (completeness)	currency (currency)			semantic consistency (concordance), plausibility (plausibility)
general data	2014	Chen, Hailey, Wang, Yu^90^	life science	accuracy (accuracy, reflecting actual sample, reliability), precision (precision), carelessness (objectivity, reflecting actual sample, reliability, under-reporting, illegible handwriting, errors in report form/errors from data entry/calculation errors), completeness (completeness), expertise (repeatability), data poisoning (objectivity)	timeliness (timeliness), age (periodicity), currency (currency, up-datedness)	Representativeness (Representativeness), granularity (granularity)	understandability (ease of understanding), feature importance (Usability, importance, relevance)	Consistency (Consistency), syntactic consistency (validity, internal consistency, external consistency, non-standardisation of vocabulary), compliance (meeting/using data standards), semantic consistency (concordance, comparability)
general data	2015	Johnson, Speedie, Simon, Kumar, Westra^96^	life science	Measurement Process (Reliability), accuracy (RelativeCorrectness, RepresentationCorrectness, RelativeCompleteness), carelessness (RepresentationIntegrity), completeness (RepresentationCorrectness, RepresentationComplete, RelativeCompleteness)	currency (RepresentationCurrent, DatasetCurrent, TaskCurrency)	variety in demographics (sufficiency), coverage (DomainCoverage, TaskCoverage)	informative missingness (DomainComplete), feature importance (relevance)	syntactic consistency (RepresentationConsistency), compliance (CodingConsistency), semantic consistency (DomainConsistency)
general data	2016	Kahn, et al.^97^	life science	completeness (completeness)			uniqueness (uniqueness)	syntactic consistency (value conformance, relational conformance, computational conformance), plausibility (plausibility)
general data	2017	Alipour^104^	life science	accuracy (accuracy, free-of-error, correctness, validity), precision (precision), carelessness (objectivity, integrity), completeness (completeness), source credibility (reputation), expertise (believability, repeatability)		Representativeness (representation), depth of data (amount of data), granularity (granularity), coverage (coverage, comprehensiveness)	understandability (understandability, interpretability, ease of understanding, readability/legibility), conciseness (conciseness), feature importance (Usability, importance, usefulness, perceived usefulness, applicable, ease of operation, relevance)	Consistency (Consistency, reliability), syntactic consistency (representational consistency), semantic consistency (concordance, comparability, integrity)
general data	2018	Corrales D, Ledezma, Corrales J^84^	non-life science	accuracy (contrast, noise, blur, occlusion, and colour degradation), noisy labels (mislabeled class), carelessness (typographical errors, spelling and format errors), outliers (outliers), completeness (missing values, missing data)	timeliness (timeliness), age (timeliness, currency)	depth of data (amount of data)	redundancy (redundancy), conciseness (high dimensionality), uniqueness (duplication, synonymous record problem)	Consistency (Consistency), syntactic consistency (consistency through integrity constraints, invalid tuples, inconsistent formatting, domain value violations), semantic consistency (semantic heterogeneity), homogeneity (heterogeneity)
general data	2018	Che, Purushotham, Cho, Sontag, Liu^135^	life science				informative missingness (informative missingness)	
general data	2019	Bloland, MacNeil^91^	life science	accuracy (Trueness), carelessness (integrity), completeness (completeness), data poisoning (integrity)	age (timeliness)	coverage (completeness)	conciseness (efficiency), feature importance (relevance)	syntactic consistency (consistency), semantic consistency (concurrence)
general data	2019	Vanbrabant, Martin, Ramaekers, Braekers^92^	life science	carelessness (Inexactness of timestamps), completeness (missing values)		granularity (imprecise data), coverage (missing entities)		syntactic consistency (typing mistake, outside domain range, inconsistent formatting, abbreviations), semantic consistency (violation of mutual dependency)
general data	2020	Bian et al.^93^	life science	accuracy (accuracy)	currency (currency)		uniqueness (uniqueness)	syntactic consistency (value conformance, relational conformance), semantic consistency (concordance, comparability), plausibility (plausibility), distribution drift (temporal plausbility)
general data	2021	Schmidt et al.^98^	life science	accuracy (accuracy, disagreement with gold standard), carelessness (inter-class/intra-class reliability), outliers (outliers, unexpected distributions, unexpected associations, unexpected data elements, unexpected data records), completeness (completeness, completeness, crude missingness), expertise (disagreement of repeated measurements)			understandability (readability/legibility), uniqueness (duplication), informative missingness (qualified missingness, non response rate, refusal rate, dropout rate, missing due to specified reason)	Consistency (Consistency), syntactic consistency (structural data set error, value format error, relational data set error, range and value violations, non-standardisation of vocabulary, data type mismatch, inhomogeneous value formats, inadmissible precision), compliance (integrity), semantic consistency (semantic consistency, accuracy/validity, contradictions, data record mismatch, data element mismatch)
general data	2021	Kim et al.^94^	life science	accuracy (accuracy), completeness (completeness)			uniqueness (uniqueness)	syntactic consistency (validity, external consistency), semantic consistency (semantic accuracy)
general data	2022	European Medicines Agency^105^	life science	Measurement Process (Reliability), accuracy (accuracy), precision (precision), completeness (completeness), traceability (traceability)	timeliness (timeliness), currency (currency)	Representativeness (Representativeness), coverage (coverage, completeness, extensiveness)	uniqueness (uniqueness), informative missingness (missingness), feature importance (relevance)	Consistency (coherence), syntactic consistency (format coherence, Structural or Relational Coherence), semantic consistency (semantic coherence), plausibility (plausibility)
general data	2023	Syed et al.^102^	life science	accuracy (accuracy, correctness, veracity), noisy labels (accurate diagnostic data), carelessness (integrity), completeness (missing data, fragmentation), data poisoning (integrity)	age (timeliness)	Representativeness (Representativeness), granularity (granularity), coverage (completeness)	uniqueness (uniqueness), feature importance (completeness, relevance)	syntactic consistency (representational consistency, validity, structuredness), compliance (integrity, standardisation), semantic consistency (semantic consistency, concordance), plausibility (plausibility), homogeneity (data variability), distribution drift (temporal variability)
general data	2023	Mashoufi, Ayatollahi, Khorasani-Zavareh, Talebi Azad Boni^101^	life science	accuracy (accuracy, correctness, validity), noisy labels (data entry error), carelessness (objectivity), completeness (completeness), source credibility (source credibility), data poisoning (objectivity)	age (currency), currency (timeliness, temporal consistency)	granularity (granularity)	feature importance (relevance)	Consistency (Consistency), syntactic consistency (conformance), semantic consistency (semantic plausibility)
general data	2023	Lewis et al.^99^	life science	accuracy (correctness), carelessness (correctness), completeness (completeness)	currency (currency)		informative missingness (bias)	semantic consistency (concordance), plausibility (plausibility)
general data	2023	Liu, Talaei-Khoei, Storey, Peng^100^	life science	accuracy (RelativeCorrectness), carelessness (correctness, misunderstanding of what an entry means, errors in report form/errors from data entry/calculation errors)	age (timeliness), currency (currency)	dataset size (completeness), coverage (completeness (coverage of baseline features or data required for a particular disease))	understandability (usability, ease of understanding), uniqueness (duplication), feature importance (relevance)	syntactic consistency (syntactic consistency, consistency through integrity constraints), distribution drift ((temporal) consistency)
general data	2023	Xu et al.^183^	non-life science	noisy labels (noisy labels), source credibility (reliability)		dataset size (dataset size)		
general data	2023	Tahar et al.^95^	life science	completeness (completeness, value completeness, item completeness)			conciseness (semantic uniqueness), uniqueness (syntactic uniqueness)	syntactic consistency (range plausibility), semantic consistency (semantic plausibility, concordance), plausibility (plausibility)
general data	2024	Declerck, Kalra, Vander Stichele, Coorevits^103^	life science	accuracy (accuracy, correctness, validity, compression/noise/blur/contrast, plausibility), completeness (completeness, capture, plausibility)	age (timeliness), currency (currency)	Representativeness (representation, relevance, precision)	understandability (understandability, contextualisation, flexibility), redundancy (redundancy), uniqueness (uniqueness), feature importance (representation)	Consistency (Consistency, stability), syntactic consistency (syntactic accuracy, conformance), semantic consistency (concordance, comparability, semantic accuracy, stability), plausibility (validity)
big data	2015	Batini, Rula, Scannapieco, Viscusi^106^	non-life science	accuracy (accuracy, semantic accuracy, source accuracy, reliability), precision (precision, accuracy deviation), noisy labels (correctness of classification), completeness (completeness, value completeness, tuple completeness, left censored, right censored, sparsity), source credibility (reputation, trustworthiness), expertise (believability), traceability (verifyability)	age (timeliness), currency (currency, temporal validity, temporal consistency)	Representativeness (Representativeness)	understandability (understandability, readability/legibility), conciseness (conciseness, closer-to-text base comprehension, Closer to situation model level comprehension, representation conc.), uniqueness (spatial redundancy, temporal redundancy), feature importance (significance, pertinence, relevance, selective)	Consistency (Consistency), syntactic consistency (syntactic accuracy, consistency through integrity constraints, consistency through edits, format consistency, domain consistency, lexical consistency, numerical consistency, range frequency, change frequency), logical consistency (logical consistency), semantic consistency (relative error consistency, coherence), plausibility (temporal consistency)
big data	2015	Cai and Zhu^108^	non-life science	accuracy (accuracy), source credibility (source credibility)	age (timeliness), currency (timeliness)	coverage (completeness)	understandability (readability), feature importance (fitness)	syntactic consistency (consistency, integrity), semantic consistency (consistency, integrity)
big data	2016	Gao, Xie, Tao^109^	non-life science	accuracy (accuracy, correctness), carelessness (correctness), completeness (completeness), source credibility (accountability)	timeliness (timeliness), currency (currency)		feature importance (Usability)	syntactic consistency (format consistency)
big data	2017	Gudivada, Apon, Ding^111^	non-life science	carelessness (gender bias), completeness (missing at random (MAR), missing completely at random (MCAR)), data poisoning (gender bias)	currency (currency)	variety in demographics (heterogeneity), variety of data sources (heterogeneity)	conciseness (dimensionality reduction), uniqueness (duplication, consistency), informative missingness (missing not at random (MNAR)), feature importance (feature selection, feature extraction)	syntactic consistency (syntactic accuracy), compliance (specifications, integrity), semantic consistency (semantic accuracy)
big data	2018	Juddoo, George, Duquenoy, Windridge^112^	life science	accuracy (accuracy), carelessness (reliability), completeness (completeness), expertise (confidence)			feature importance (usefulness)	syntactic consistency (consistency), semantic consistency (consistency), plausibility (validity)
big data	2019	Ijab, Mat Surin, Mat Nayan^113^	non-life science	accuracy (accuracy, errors), source credibility (source credibility, reliability, authenticity)	timeliness (timeliness)	variety (variety)	understandability (usability, readability, clarity, misleading)	syntactic consistency (validity), semantic consistency (concordance, agreement, consistency)
big data	2020	Eder, Shekhovtsov^107^	life science	accuracy (accuracy), completeness (completeness), source credibility (reliability)	age (timeliness)	granularity (precision (no. of digits))	feature importance (reliability)	compliance (compliance), semantic consistency (semantic consistency)
big data	2020	Ramasamy, Chowdhury^110^	non-life science	source credibility (source credibility, trustworthiness), traceability (Pedigree/Lineage)		dataset size (volume)	redundancy (redundancy)	semantic consistency (cohesion)
Maccuracy (accuracy, free-of-error, correctnessL data	1993	Bansal, Kauffman, Weitz^130^	non-life science	carelessness (typing error (by white noise), measurement of subjective data (e.g., about consumer sentiment) -> as white noise, inaccurate estimations (e.g., from forecasts))				
ML data	2000	Michel, Zernikow, Wichert^125^	life science			dataset size (quantity), coverage (completeness)		
ML data	2011	Blake, Mangiameli^133^	non-life science	accuracy (accuracy), completeness (completeness)	age (timeliness)			syntactic consistency (consistency)
ML data	2011	Zhou, Wu^129^	non-life science	accuracy (adding random errors)		dataset size (dataset size)		
ML data	2013	Twala^131^	non-life science	precision (noisy attributes), noisy labels (noisy targets), carelessness (noisy attributes), outliers (outliers)				
ML data	2015	Ghotra, McIntosh, Hassan^128^	non-life science	accuracy (errors), completeness (missing values)		variety of data sources (bias)	uniqueness (identical cases)	semantic consistency (conflicting data, inconsistent cases), plausibility (plausibility)
ML data	2015	Sukhbaatar, Bruna, Paluri, Bourdev, Fergus^157^	non-life science	noisy labels (noisy labels, label flips, outliers)				
ML data	2015	Masko, Hensman^140^	non-life science			target class balance (imbalance)		
ML data	2016	Dodge, Karam^160^	non-life science	accuracy (compression/noise/blur/contrast)				
ML data	2016	Karahan, et al.^161^	non-life science	accuracy (contrast, noise, blur, occlusion, and colour degradation)				
ML data	2017	Sun, Shrivastava, Singh, Gupta^146^	non-life science	noisy labels (noisy labels)		dataset size (dataset size), coverage (number of classes)		
ML data	2017	Barakat et al.^126^	life science	completeness (missing at random (MAR), missing completely at random (MCAR), missing values)		dataset size (dataset size)	informative missingness (missing not at random (MNAR))	
ML data	2017	Snodgrass, Summerville, Ontañón^190^	non-life science			variety in demographics (variety of subjects), depth of data (amount of data)		
ML data	2018	Rolnick, Veit, Belongie, Shavit^169^	non-life science	noisy labels (noisy labels, uniform label noise, structured label noise)		dataset size (dataset size)		
ML data	2018	Wang et al.^170^	non-life science	noisy labels (noisy labels)				
ML data	2018	Nuha, Afiahayati^147^	non-life science			dataset size (dataset size)		
ML data	2018	Buda, Maki, Mazurowski^141^	non-life science			target class balance (target class balance)		
ML data	2019	Pei, Huang, Zou, Zhang, Wang^162^	non-life science	accuracy (hazy images, underwater images, motion-blurred images, fish-eye-images)				
ML data	2019	Schnabel, Matzka, Stellmacher, Patzold, Matthes^163^	non-life science	accuracy (anonymization (blurring, mask-out, realistic anonymization...))				
ML data	2019	He, Yu, Wang, Li, Chen^159^	non-life science	noisy labels (quality of label), data poisoning (dataset contamination)		dataset size (dataset size), target class balance (dataset equilibrium)		
ML data	2019	Peterson, Battleday, Griffiths, Russakovsky^171^	non-life science	noisy labels (human uncertainty)				homogeneity (out of distribution)
ML data	2019	Ismail Fawaz, Forestier, Weber, Idoumghar, Muller^136^	non-life science	data poisoning (adversarial attacks/time series perturbation)				
ML data	2019	Habib, Karmakar, Yearwood^137^	life science			variety of data sources (inter-database testing)		
ML data	2019	Johnson, Khoshgoftaar^142^	non-life science			target class balance (target class balance)		
ML data	2019	Ovadia et al.^145^	non-life science					distribution drift (distribution drift)
ML data	2020	Karimi, Dou, Warfield, Gholipour^172^	life science	accuracy (error/noise in labels generated by an algorithm), noisy labels (systematic error by a human annotator, label noise due to inter-observer variability)				
ML data	2020	Taran, Gordienko, Rokovyi, Alienin, Stirenko^173^	non-life science	noisy labels (label coarsity)				
ML data	2020	Johnson, Khoshgoftaar^115^	non-life science			target class balance (target class balance)		
ML data	2020	Sahu, Mao, Davis, Goulart^116^	non-life science			target class balance (imbalance)	uniqueness (feature reduction)	syntactic consistency (normalisation)
ML data	2020	Cao, Hu, Gao, Wang, Ming^114^	non-life science				uniqueness (rank)	
ML data	2021	Hong, Shen^148^	life science	accuracy (adding white noise)		dataset size (dataset size)		
ML data	2021	Benedick, Robert, Traon^134^	non-life science	accuracy (swapping perturbation), completeness (dropping perturbation)				
ML data	2021	Zhong, et al.^164^	non-life science	accuracy (device condition), precision (signal variation), carelessness (user-controlled settings)				
ML data	2021	Wesemeyer, Jauer, Deserno^158^	life science	noisy labels (annotation quality)		dataset size (quantity)		
ML data	2021	Volkman et al.^174^	non-life science	noisy labels (high quality annotations)				
ML data	2021	Barragán-Montero et al.^154^	life science	noisy labels (variety)		dataset size (dataset size)		
ML data	2021	Motamedi, Sakharnykh, Kaldewey^155^	non-life science	noisy labels (Dataset Investigation)		dataset size (GAN-based sample synthesis), target class balance (Class Imbalance Resolution)	uniqueness (Duplicate Detection and Elimination)	
ML data	2021	Qi, Wang, Wang^117^	non-life science	completeness (missing data)				semantic consistency (violations of functional dependencies, conflicting data)
ML data	2021	Eid et al.^191^	life science			variety of data sources (variety of data sources, bias)		
ML data	2021	Bailly et al.^123^	life science			dataset size (dataset size)		
ML data	2021	Althnian et al.^124^	life science			dataset size (dataset size)		
ML data	2021	Ito, Saito, Ueno, Homma^138^	non-life science			dataset size (dataset size), target class balance (imbalance)		
ML data	2021	Li, Chao^149^	non-life science			dataset size (quantity)	feature importance (information value)	
ML data	2021	Li, Zhao, Caragea^184^	non-life science			dataset size (multi-dataset training)		
ML data	2021	Li, Yang, Wen^150^	non-life science			target class balance (target class balance)	redundancy (redundancy), feature importance (informative sample)	
ML data	2022	Fan, Shi^151^	life science	accuracy (adding random errors)		dataset size (quantity)		
ML data	2022	Wei et al.^175^	non-life science	noisy labels (noisy labels)				
ML data	2022	Schmarje et al.^177^	non-life science	noisy labels (noisy labels)				
ML data	2022	Ma, Ushiku, Sagara^176^	non-life science	noisy labels (annotation quality)				
ML data	2022	Tran, Chen, Bhuyan, Ding^120^	non-life science	noisy labels (labelling accuracy), source credibility (source credibility, reputation, trustworthiness, reliability)	age (timeliness, currency)	variety (variety), coverage (coverage, comprehensiveness)	uniqueness (duplication, class overlap), feature importance (relevance, fit for purpose)	Consistency (Consistency), syntactic consistency (syntactic accuracy, validity, integrity), semantic consistency (semantic accuracy, integrity), plausibility (validity)
ML data	2022	Wang, Gao, Li, Hu^181^	non-life science	noisy labels (Noisy Location Annotation)				
ML data	2022	Jouseau, Salva, Samir^119^	non-life science	outliers (outliers), completeness (missing values)			uniqueness (exact and partial duplicates), informative missingness (missing values)	syntactic consistency (domain value violations)
ML data	2022	Nikolados, Wongprommoon, Aodha, Cambray, Oyarzùn^188^	life science			variety of data sources (heterogeneity), dataset size (dataset size)		
ML data	2022	Lake, Tsai^122^	non-life science			dataset size (dataset size), target class balance (target class balance)		
ML data	2022	Derry, Carpenter, Altman^187^	life science			dataset size (more training data), granularity (downsampling)	feature importance (use of different experimental methods)	
ML data	2022	Guo et al.^192^	life science					distribution drift (distribution shift)
ML data	2023	Zhang, Singh, Ghassemi, Joshi^139^	non-life science					distribution consistency (distribution drift)
ML data	2023	Güneş et al.^168^	life science	accuracy (correctness, blur, overexposure, contrast, brightness, hue, sharpness, saturation and compression artefacts, artefacts)				
ML data	2023	Ranjan,Sharrer,Tsukuda, Good^152^	non-life science	accuracy (lighting condition), precision (augmentation (mixup/brightness/exposure/saturation/noise/blur/cutout/mosaic))		dataset size (dataset size, augmentation)		
ML data	2023	Jaspers et al.^166^	life science	accuracy (blur, overexposure, contrast, brightness, hue, sharpness, saturation and compression artefacts)				
ML data	2023	Hukkelås, Lindseth^165^	non-life science	accuracy (anonymization (blurring, mask-out, realistic anonymization...))				
ML data	2023	Whang, Roh, Song, Lee^182^	non-life science	precision (augmentation (mixup/brightness/exposure/saturation/noise/blur/cutout/mosaic), noisy features (image blur/colour noise)), noisy labels (noisy labels), carelessness (racial bias, fairness), completeness (missing values, missing labels), data poisoning (data poisoning, label flipping, racial bias, fairness)			informative missingness (informative missingness)	
ML data	2023	Hu, Wang^118^	non-life science	precision (uncertainty), noisy labels (outliers)			uniqueness (uniqueness)	
ML data	2023	Costa, Silva, Costa, Ribeiro^179^	non-life science	noisy labels (noisy labels)				
ML data	2023	Shimizu, Wakabayashi^185^	life science	noisy labels (label redundancy)		dataset size (budget for crowdsourcing)		
ML data	2023	Bai et al.^143^	life science	noisy labels (interobserver variability)		variety of data sources (variety of data sources)	feature importance (feature importance)	
ML data	2023	Agnew et al.^178^	non-life science	noisy labels (Annotation Quality)				
ML data	2023	Radliński^127^	non-life science	completeness (completeness, missing values)				syntactic consistency (validity, integrity)
ML data	2023	Sha, Gašević, Chen^121^	non-life science			Representativeness (distribution bias (imbalance of feature distribution within target classes))		
ML data	2023	Zengin, Yenisey, Kutlu^186^	non-life science			dataset size (dataset size)		homogeneity (different targets)
ML data	2023	Wang, Jackson^189^	life science			dataset size (sample size), granularity (detection limit)		
ML data	2023	Pan, Xie, Zhao^144^	non-life science			target class balance (target class balance)		homogeneity (interclass ambiguity, out of distribution)
ML data	2024	Deshsorn, Lawtrakul, Iamprasertkun^132^	non-life science	accuracy (adding random errors), precision (noisy features (image blur/colour noise)), noisy labels (noisy targets)				
ML data	2024	Lee, You^167^	non-life science	accuracy (blurring, pixelation, distortion), completeness (mask-out (pixel))				
ML data	2024	Xu, Yue, Liu, Chen^156^	non-life science	noisy labels (annotation quality)		dataset size (dataset size)		
ML data	2024	Cui et al.^180^	non-life science	noisy labels (left-shifted, right-shifted, upward-shifted, downward-shifted, overestimated, underestimated, incorrect annotations), completeness (missing labels)				
ML data	2024	Vilaça, Viana, Carvalho, Andrade^153^	life science			variety in demographics (variety of subjects), dataset size (dataset size)	feature importance (entropy/diversity)	

It seems that with 461 extracted terms, we are beyond a saturation point of finding new data quality dimensions. From a certain point on, more synonyms do not uncover new concepts. From a bias assessment point of view, it is possible that the literature that investigates effects of data quality on ML could be skewed towards investigating and reporting dimensions with bigger effects. The risk of missing out on vocabulary due to this is mitigated by the inclusion of broad theoretical frameworks in our literature corpus.

Thorough discussion of all authors about underlying concepts and definitions of the subdimensions resulted in hierarchically grouping these into dimensions and the dimensions into clusters. In parallel to this grouping, all authors reached consensus on definitions for dimensions and subdimensions of the METRIC-framework. The definitions were adopted from a recent data quality glossary^197^ if they existed there and met our understanding of the vocabulary in the given context of medical training data. If necessary, we included definitions given by Wang et al.^57^ in a second iteration. If none of these two sources suggested an appropriate definition, we captured the meaning of the desired term on the basis of the literature corpus and thus determined its definition in the context of medical training data (see Tables 1–6).

### Reporting summary

Further information on research design is available in the Nature Research Reporting Summary linked to this article.

### Supplementary information

Reporting Summary

Supplementary Data 1

Supplementary Data 2

PRISMA Checklist

## Data Availability

All data utilised in this study is available. The literature database that serves as a basis for this systematic review is provided in Supplementary Data 1. The extracted data quality vocabulary from the literature database that serves as a basis for the METRIC-framework is provided in Supplementary Data 2.

## Code availability

All code utilised for this study is available at https://github.com/danielschw188/ReviewPaper_DataQualityForMLinMedicine.
