# Supplementary material for: The METRIC-framework for assessing data quality for trustworthy AI in medicine: a systematic review
Source: NPJ Digit Med. 2024 Aug 3;7:203. doi: 10.1038/s41746-024-01196-4 (PMC11297942; doi:10.1038/s41746-024-01196-4)
Supplement: Supplementary file 1 — Reporting Summary [file 41746_2024_1196_MOESM1_ESM.pdf]

nature portfolio

Corresponding author(s): Daniel Schwabe

Last updated by author(s): May 3, 2024

Reporting Summary

Nature Portfolio wishes to improve the reproducibility of the work that we publish. This form provides structure for consistency and transparency in reporting. For further information on Nature Portfolio policies, see our Editorial Policies and the Editorial Policy Checklist.

Statistics

For all statistical analyses, confirm that the following items are present in the figure legend, table legend, main text, or Methods section.

n/a	Confirmed	
☐	☒	The exact sample size (n) for each experimental group/condition, given as a discrete number and unit of measurement
☒	☐	A statement on whether measurements were taken from distinct samples or whether the same sample was measured repeatedly
☒	☐	The statistical test(s) used AND whether they are one- or two-sided<br>*Only common tests should be described solely by name; describe more complex techniques in the Methods section.*
☒	☐	A description of all covariates tested
☒	☐	A description of any assumptions or corrections, such as tests of normality and adjustment for multiple comparisons
☒	☐	A full description of the statistical parameters including central tendency (e.g. means) or other basic estimates (e.g. regression coefficient) AND variation (e.g. standard deviation) or associated estimates of uncertainty (e.g. confidence intervals)
☒	☐	For null hypothesis testing, the test statistic (e.g. F, t, r) with confidence intervals, effect sizes, degrees of freedom and P value noted<br>*Give P values as exact values whenever suitable.*
☒	☐	For Bayesian analysis, information on the choice of priors and Markov chain Monte Carlo settings
☒	☐	For hierarchical and complex designs, identification of the appropriate level for tests and full reporting of outcomes
☒	☐	Estimates of effect sizes (e.g. Cohen's d, Pearson's r), indicating how they were calculated

Our web collection on statistics for biologists contains articles on many of the points above.

Software and code

Policy information about availability of computer code

Data collection

Databases "Web of Science" (WoS), "PubMed" (PM) and "ACM Digital Library" (ACM) were searched with keywords on 12th April 2024.
Specification for searches:
- WoS: Advanced search, post search string into query box
- PM: Advanced Search, post search string into query box
- ACM:
    - Field "Search items from": "The ACM Guide to Computing Literature"
    - Field "Search Within": Perform two separate searches:
        - 1. Choose "Title", post search string into query box
        - 2. Choose "Abstract", post search string into query box

Search strings are included in supplementary file. Raw results were downloaded into 9 distinct files (WoS: 4, PM: 1, ACM: 4). WoS enabled downloads of 1000 results per file (3165 total results). Only the first 1000 results can be downloaded from ACM per search. Thus, the full search string had to be divided into parts that did not have more than 1000 results on ACM per search.
Raw results were concatenated (full join). Duplicates with respect to DOIs and exact title matches were removed. The code used for these two steps is available on Github (https://github.com/danielschw188/ReviewPaper_DataQualityForMLinMedicine).
Results were then transferred to an Excel list which represents the literature database for this review.

Data analysis

All code utilised in this study is available. For the generation of Figure 2, code was written in R. It is available on Github: https://github.com/danielschw188/ReviewPaper_DataQualityForMLinMedicine.

For manuscripts utilizing custom algorithms or software that are central to the research but not yet described in published literature, software must be made available to editors and reviewers. We strongly encourage code deposition in a community repository (e.g. GitHub). See the Nature Portfolio guidelines for submitting code & software for further information.

Data

Policy information about availability of data

All manuscripts must include a data availability statement. This statement should provide the following information, where applicable:

- Accession codes, unique identifiers, or web links for publicly available datasets
- A description of any restrictions on data availability
- For clinical datasets or third party data, please ensure that the statement adheres to our policy

All data utilised in this study is available. The literature database that serves as a basis for this systematic review is provided in the supplementary file "METRIC_LiteratureDatabase.xlsx". The extracted data quality vocabulary from the literature database that serves as a basis for the METRIC-framework is provided in the supplementary file "METRIC_ExtractedDataQualityVocabulary.xlsx".

Research involving human participants, their data, or biological material

Policy information about studies with human participants or human data. See also policy information about sex, gender (identity/presentation), and sexual orientation and race, ethnicity and racism.

Reporting on sex and gender	No sex or gender based information has been collected.
Reporting on race, ethnicity, or other socially relevant groupings	No socially relevant categorization variables were used in this study.
Population characteristics	The study does not contain human research participants.
Recruitment	No human participants were recruited in this study. The selected studies to be considered for this systematic review were selected by carefully chosen search string and obtained through an online database search (see Methods section).
Ethics oversight	The study follows the PRISMA guidelines for systematic reviews.

Note that full information on the approval of the study protocol must also be provided in the manuscript.

Field-specific reporting

Please select the one below that is the best fit for your research. If you are not sure, read the appropriate sections before making your selection.

☒ Life sciences ☐ Behavioural & social sciences ☐ Ecological, evolutionary & environmental sciences

For a reference copy of the document with all sections, see nature.com/documents/nr-reporting-summary-flat.pdf

Life sciences study design

All studies must disclose on these points even when the disclosure is negative.

Sample size	The systematic review considers 5408 studies, given by the number of unique search results.
Data exclusions	No study was excluded from screening process. For extracting a literature corpus from the literature database, clear eligibility criteria (inclusion/exclusion criteria) were established and are described in the Methods section.
Replication	Replication of results is possible by utilizing the same data collections steps described. Small deviations might occur if records should be deleted from the online databases since the original search. In addition, the current version of the ACM search engine does not enable specifying an exact date of results, only a specific month. Our search was performed on 12th April 2024 meaning a future search on ACM with the filter "April 2024" will contain additional results from the month of April 2024 that were not yet published during the original search.
Randomization	List of search results were downloaded from online databases, concatenated and duplicates removed. No further randomization was necessary. Records were seen by two authors each. Thus, no random allocation of studies was performed.
Blinding	Reviewers were blinded from other reviewers' decisions.

Reporting for specific materials, systems and methods

We require information from authors about some types of materials, experimental systems and methods used in many studies. Here, indicate whether each material, system or method listed is relevant to your study. If you are not sure if a list item applies to your research, read the appropriate section before selecting a response.

Materials & experimental systems

n/a	Involved in the study
☒	☐ Antibodies
☒	☐ Eukaryotic cell lines
☒	☐ Palaeontology and archaeology
☒	☐ Animals and other organisms
☒	☐ Clinical data
☒	☐ Dual use research of concern
☒	☐ Plants

Methods

n/a	Involved in the study
☒	☐ ChIP-seq
☒	☐ Flow cytometry
☒	☐ MRI-based neuroimaging

Plants

Seed stocks	*Report on the source of all seed stocks or other plant material used. If applicable, state the seed stock centre and catalogue number. If plant specimens were collected from the field, describe the collection location, date and sampling procedures.*
Novel plant genotypes	*Describe the methods by which all novel plant genotypes were produced. This includes those generated by transgenic approaches, gene editing, chemical/radiation-based mutagenesis and hybridization. For transgenic lines, describe the transformation method, the number of independent lines analyzed and the generation upon which experiments were performed. For gene-edited lines, describe the editor used, the endogenous sequence targeted for editing, the targeting guide RNA sequence (if applicable) and how the editor was applied.*
Authentication	*Describe any authentication procedures for each seed stock used or novel genotype generated. Describe any experiments used to assess the effect of a mutation and, where applicable, how potential secondary effects (e.g. second site T-DNA insertions, mosiacism, off-target gene editing) were examined.*
